# Inferring the Chemotactic Strategy of *P. putida* and *E. coli* Using Modified Kramers-Moyal Coefficients

**DOI:** 10.1371/journal.pcbi.1005329

**Published:** 2017-01-23

**Authors:** Oliver Pohl, Marius Hintsche, Zahra Alirezaeizanjani, Maximilian Seyrich, Carsten Beta, Holger Stark

**Affiliations:** 1 Institute of Theoretical Physics, Technical University Berlin, Berlin, Germany; 2 Institute of Physics and Astronomy, University of Potsdam, Potsdam, Germany; University of Reading, UNITED KINGDOM

## Abstract

Many bacteria perform a run-and-tumble random walk to explore their surrounding and to perform chemotaxis. In this article we present a novel method to infer the relevant parameters of bacterial motion from experimental trajectories including the tumbling events. We introduce a stochastic model for the orientation angle, where a shot-noise process initiates tumbles, and analytically calculate conditional moments, reminiscent of Kramers-Moyal coefficients. Matching them with the moments calculated from experimental trajectories of the bacteria *E. coli* and *Pseudomonas putida*, we are able to infer their respective tumble rates, the rotational diffusion constants, and the distributions of tumble angles in good agreement with results from conventional tumble recognizers. We also define a novel tumble recognizer, which explicitly quantifies the error in recognizing tumbles. In the presence of a chemical gradient we condition the moments on the bacterial direction of motion and thereby explore the chemotaxis strategy. For both bacteria we recover and quantify the classical chemotactic strategy, where the tumble rate is smallest along the chemical gradient. In addition, for *E. coli* we detect some cells, which bias their mean tumble angle towards smaller values. Our findings are supported by a scaling analysis of appropriate ratios of conditional moments, which are directly calculated from experimental data.

## Author Summary

The movement strategies of bacteria have received increasing attention over the past decade, in particular with respect to the tracking of individual cells and the mathematical description of the resulting trajectories. Bacteria typically move in almost straight runs interrupted by sharp turning events (run-and-tumble). In order to characterize their motion on a single cell level, the tumble events in individual trajectories have to be identified. Traditionally, tumble recognition relies on threshold values that are applied to the swimming speed and the reorientation angle. They are chosen in an ad hoc fashion and introduce a certain degree of arbitrariness to the results of statistical motion analyses. Here, we propose a new stochastic model for the orientation angle of a bacterium and formulate conditonal moments, which we determine both in theory and from experimental trajectories. This provides an alternative way of quantifying the bacterial run-and-tumble strategy and of recognizing tumble events. Our approach no longer relies on arbitrarily chosen segmentation thresholds and rigorously quantifies the uncertainty in tumble recognition. We successfully apply our method not only to the paradigmatic case of *E. coli* but also to trajectories of the soil bacterium *Pseudomonas putida*, demonstrating that our approach provides a novel way to reliably characterize the tumbling statistics and chemotaxis strategies of bacterial swimmers across different species.

## Introduction

Taxis refers to the ability of microorganisms to sense and move along the gradient of an external stimulus or field [1]. The most prominent example is chemotaxis, where the gradient is formed by the density of a chemical species [2]. Many microorganisms perform chemotaxis [2–5] but also synthetic swimmers are able to swim along chemical gradients [6–9]. Other forms of taxis, such as gravitaxis [10, 11], rheotaxis [12], magnetotaxis [13–16], or thermotaxis [17–19], were investigated for both biological and artificial microswimmers.

The chemotactic behavior of bacteria is a fascinating topic to study since not only the underlying biochemical signaling pathway but also their active random walk needs to be considered [20–23]. A very common moving pattern for bacteria is the so-called run-and-tumble random walk. Quantitatively, it was first studied by Berg and Brown [2] in the early seventies revealing the distribution of tumble angles and the tumble rate. In the same paper the authors also showed that *E. coli* decreases its tumble rate when moving along a chemical gradient. Later, bacterial response to chemical landscapes changing in time and space was studied [24, 25]. In Ref. [26] a bias in the tumble angle was reported, i.e., the mean reorientation angle during tumbling is smaller when moving along a chemical gradient than against it. To the best of our knowledge, this result has not been verified in other experiments. In this paper we will provide further evidence for such an angle bias.

To separate runs from tumbles in bacterial trajectories, a computer algorithm called *tumble recognizer* has been used [2, 25]. It recognizes tumbles along the bacterial trajectories based on changes in moving direction but also in speed. When these changes are large compared to a set of threshold parameters, a tumble is detected. The parameters are chosen such that the automatized tumble recognition agrees with visual inspections of the trajectories. Hence, there is no general rule of choosing them.

Another method to quantify the tumble behavior of bacteria is the technique of parameter inference. Parameters used in theoretical models are determined by appropriate numerical optimization procedures such that experimental data are best reproduced within the model. Compared to tumble recognizers an a prioiri definition of threshold parameters is not needed. Recently, the technique of Bayesian inference has been applied to determine the chemotactic response function of *E. coli* [25] as well as distributions of reorientation angles and speed changes [27]. In both cases, the desired model parameters are obtained by maximizing a likelihood function, which contains the data of all recorded trajectories. Thus, the optimization poses a complex numerical task [28].

In this work, we propose a different approach to infer the statistics of tumbling and chemotaxis from experimental data. It has the advantage that it greatly reduces the complexity of the optimization and it also operates without any predefined parameters. Only a familiy of tumble angle distributions needs to be specified a priori. As key tool to shrink the extensive data amount from recorded experimental trajectories, our approach uses a special form of conditional moments (CMs) [29, 30], which we introduce in close analogy to Kramers-Moyal coefficients of stochastic processes [31]. The CMs are calculated from a minimal stochastic model of run-and-tumble motion, where tumble events are initiated by shot-noise, and matched to the moments obtained from experimental trajectories. Thereby, we not only infer parameters determining tumbling but also the rotational diffusion constant. Also, by analyzing appropriate ratios of the experimental CMs, we are able to draw direct conclusions on chemotactic strategies without fitting any parameters. Thus, we are able to verify the results obtained from parameter inference.

Kramers-Moyal coefficients were used before to analyze experimental data in order to distinguish the drift component from diffusion in the stochastic dynamic behavior of biological organisms [32]. For example, random turns of locust swarms [33], moving patterns of the amoeba *Dictyostelium discoidum* [5], or gene regulations [34] were studied with this approach. In our case, the dynamics is clearly non-Brownian due to the shot noise modeling tumble events. Thus, more than the first two Kramers-Moyal coefficients become relevant and can be considered when evaluating experimental data.

In the following, we apply our method of CMs to experimentally recorded trajectories of the bacteria *E. coli* and *P. putida* moving in gradients of a chemoattractant. Our goal is to demonstrate that with this method we are able to characterize the bacterial chemotactic response by extracting the relevant parameters of the distribution of tumble angles, the mean tumble rate, and the rotational diffusion coefficient. We start with introducing our shot-noise model for the run-and-tumble motion, explain the method of CMs, and how they are determined from experimental trajectories, and finally give experimental details about cell culture, microfluidic setup, and cell tracking.

In the results section we first test our method against a conventional tumble recognizer and infer the relevant parameters without considering the direction of the chemical gradient. We also use these paramters to define a novel tumble recognizer. Then, we condition the CMs on the bacterial swimming direction to characterize bacterial chemotaxis. We quantitatively confirm the traditional picture that the mean tumble rate decreases when *E. coli* swims along the chemical gradient but also find that the mean tumble angle for some of the bacteria is clearly biased to smaller values in this swimming direction. Such an angle bias was predicted in Ref. [35] and also found in Ref. [26]. Finally, we apply our method to the bacterium *P. putida*, detect the classical chemotaxis strategy, and quantify it.

**Fig 1:**
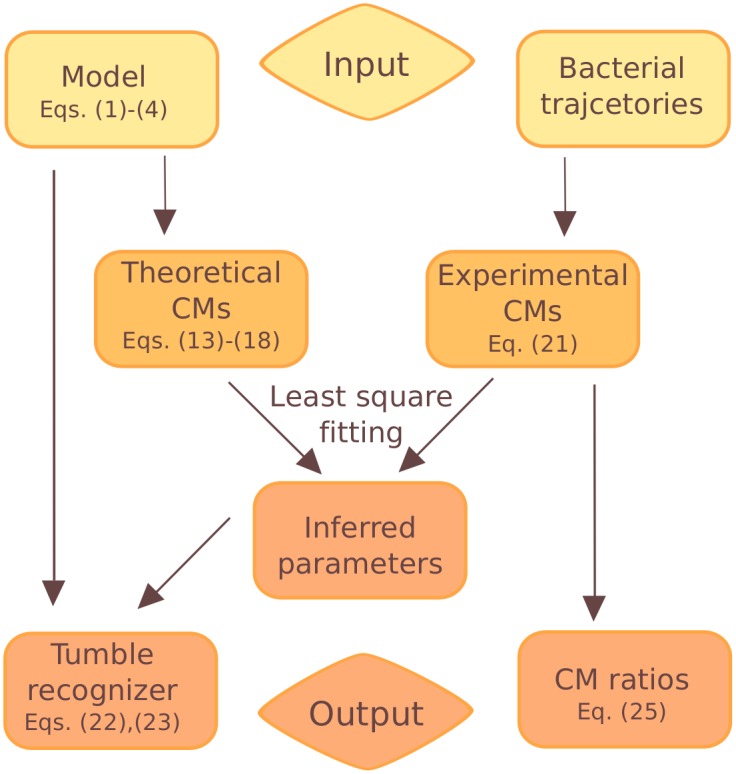
Flow diagram of the CM method. As input one provides the model of bacterial motion summarized in Eqs (1)–(4) and a sufficient number of experimental trajectories. Then, the theoretical CMs **m**_theo_(*θ*,**p**) are calculated from the model as a function of the parameters **p** and the current orientation angle *θ* [see Eqs (13)–(18)] The experimental CMs **m**_exp_(*θ*) are determined using Eq (21). Matching these moments with a least square fit yields as an output the parameters of the model: **p**(*θ*) = arg min_**p**_|**m**_exp_(*θ*) − **m**_theo_(**p**)|^2^. Furthermore, starting from the model and using the inferred parameters, we introduce a new tumble recognizer and test it against a heuristic tumble recognizer. Finally, the CM ratios are obtained directly from the experimental CMs and are used to identify the angle bias.

An overview of our method of conditional moments is depicted in the flow diagram of Fig 1 and explained in the caption. It can also be used as a guide through the article. In the first large section, we explain the theoretical modeling of bacterial motion, introduce and calculate the conditional moments, provide the tumble angle distribution for *E. coli*, and give details of the experiments. Furthermore, we provide information on the computer code we developed to implement the method of conditional moments, and which we made freely available on github. After the large results section we close with a discussion.

## Models

### Shot noise model for run-and-tumble motion

A typical moving pattern for bacteria, such as *E. coli*, is the so-called “run-and-tumble” random walk [23]. It consists of a running and a tumble phase. During the first phase the bacterium moves forward on a nearly straight path, only rotational thermal noise affects its persistence. During the tumble state the bacterium reduces its velocity and reorients rapidly in a new direction with a reorientation angle *β*. To account for these two systematically different types of motion, we use two stochastic processes *q* and *ξ*, which govern the angular dynamics in the following overdamped Langevin equations:

$$\dot{r}\left(t\right)=v\left(t\right)e\left(t\right),$$ (1)

$$\dot{\Theta }\left(t\right)=q\left(t\right)+\xi \left(t\right)\;.$$ (2)

Here, Θ is the bacterium’s orientation angle, **r** the two-dimensional position vector, **e** = (cos Θ, sin Θ) is the orientation vector of the bacterium, and *v*(*t*) the swimming velocity, which strongly decreases during a tumble event. In this article we focus on the angular dynamics and do not specify *v*(*t*) further. *ξ* is a white-noise process, which accounts for rotational thermal noise due to the ambient fluid. As usual, it is fully characterized by its mean value, 〈*ξ*(*t*)〉 = 0, and the correlation function 〈*ξ*(*s*)*ξ*(*t*)〉 = 2*D*_rot_, where *δ*(*t* − *s*) is the delta function. The tumble events are modeled by a shot-noise process [36, 37],

$$q(t)=\sum_{i=1}^{N^{\lambda }}\beta_{i}\delta \left(t-t_{i}\right),$$ (3)

which is a train of *N*^λ^ delta spikes with tumble amplitudes *β*_*i*_. The tumbles obey a Poisson distribution. They occur randomly at each time step Δ*t* with probability Δ*t*λ(*t*), where λ(*t*) is the time-dependent tumble rate. The assumption of a Poisson distribution implies exponentially distributed run times between the shots. However, as we show in S6 Text our method can be applied without modification for all run time distributions with finite first moment. The random variables *β*_*i*_ ∈ [−*π*, *π*] represent the reorientation angles during the tumble events. They are symmetrically distributed about *β*_*i*_ = 0, i.e., rightward and leftward tumbling is equally probable. The probability distribution *P*(|*β*|) varies with the particular organisms studied [23, 38]. We specify it for *E. coli* in Eq (19) and for *P. putida* in Eq (26).

Eq (2) can be integrated to

Θ(t)=N(t)+B(t), (4)

where *N* represents the inhomogeneous Poisson process with rate function λ(*t*) for the tumble events. The rotational Brownian motion *B* is implemented such that for each time step Δ*t*, the angular step is taken from a normal distribution $P\left[B\left(t+\Delta t\right)-B\left(t\right)\right]=P\left(dB\right)=N\left(0,2D_{\text{rot}}\Delta t\right)$, with zero mean and variance 2*D*_rot_Δ*t*. In the following we will use *N* and *B* to indicate the stochastic processes instead of *q* and *ξ*.

For completeness we give the tumble rate

$$\lambda \left(t\right)=\lambda \left[r\left(t\right)\right]≔\lambda_{\text{equ}}-\int_{-\infty }^{t}R\left(t-t^{\prime }\right)c\left(r\left(t^{\prime }\right)\right)dt^{\prime }\;,$$ (5)

which is typically modeled as a convolution of the chemotactic field experienced along the bacterial path **r**(*t*) and the so-called chemotactic response function. Its precise shape has extensively been discussed in the literature, e.g., in Refs. [2, 25]. We will not use Eq (5) in our method of conditional moments but refer to it to interpret our results.

### Conditional moments

Our model implements a time-continuous realization of bacterial run-and-tumble motion and effectively separates two time scales, one for running and another for tumbling. We will use it to infer the relevant parameters of bacterial motion from experimental trajectories including their tumbling events. Note that our modeling of tumbles as delta spikes is an approximation that neglects the finite tumble time. In the discussion section we suggest an extension of our model equations by including temporal speed variations in combination with a finite tumble time. In the current work, we explicitly excluded experimental trajectories, where the cells tumble for very long times, so that our current approach is valid. Manual or automized filtering procedures are commonly used for the analysis of bacterial trajectories [2, 5, 25, 27]

As the key tool of our inference, we define the *n*-th absolute conditional moment (CM) of our stochastic process Θ(*t*) for a given finite time step Δ*t* as

$$m_{\Delta t}^{n}\left(\theta \right)\left[\Theta \right]≔\left\langle \left.\frac{{\left|\Theta \left(t+\Delta t\right)-\Theta \left(t\right)\right|}_{a}^{n}}{\Delta t}\;\right|\;\Theta \left(t\right)=\theta \right\rangle .$$ (6)

**Fig 2:**
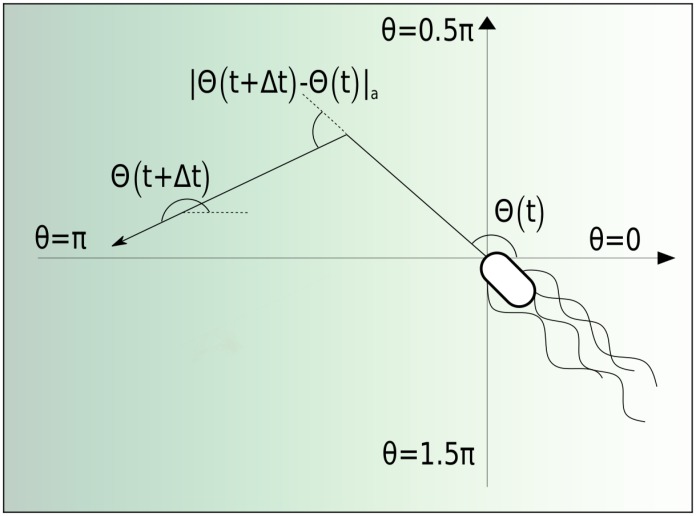
Schematics of a bacterial tumble event. *E. coli* moves in direction Θ(*t*), tumbles at time *t* + Δ*t*, and moves in the new direction Θ(*t* + Δ*t*). Thus, the turning angle becomes |Θ(*t* + Δ*t*) − Θ(*t*)|_*a*_.

With |…|_*a*_ we always select the absolute value of the changing angle, which is smaller than *π*: |*α*|_*a*_ = min(|*α*|, 2*π* − |*α*|). Furthermore, Δ*t* is non-zero and chosen such that it is much larger than the mean tumbling time. In Fig 2 we show part of a bacterial trajectory and define the relevant orientation angles.

We condition the moments on the prior moving direction Θ(*t*) = *θ* such that they become functions of *θ*. In the presence of a nutrient gradient the moments depend on *θ* because this angle indicates whether the cell swims up or down the gradient. The CMs are reminiscent of the Kramers-Moyal coefficients $m^{n}\left(x\right)\left[X\right]≔\lim_{\Delta t\to 0}\left\langle \frac{{\left[X\left(t+\Delta t\right)-X\left(t\right)\right]}^{n}}{\Delta t}|X\left(t\right)=x\right\rangle$. However, they are defined with absolute values and Δ*t* is finite. Note, for Δ*t* → 0 the first absolute CM of Brownian motion diverges as $1/\sqrt{\Delta t}$, as demonstrated below. Furthermore, without taking the absolute value the odd moments vanish because both, tumbling and rotational diffusion, occur with equal probability to the left or right. Therefore, to have additonal moments available to be fitted to the experimental data in our inference procedure, we use the absolute value in the definition (6) of the CMs.

Typically, with the first two moments one can distinguish between the deterministic and stochastic terms in stochastic differential equations [39]. Analyzing the Kramers-Moyal coefficients, one can explicitly reproduce the drift and diffusion functions, which govern the dynamics of various biologic systems. However, for non-Brownian stochastic processes, moments with *n* larger than 1 or 2 do not necessarily vanish. This is indeed the case for our shot-noise process.

#### Evaluation of the CMs

In the following we explicitly evaluate the CMs for even and odd power *n*. For readers not interested in the detailed calculations, the final results are given in Eqs (13)–(18). Besides the mean tumble rate λ, the rotational diffusion coefficient *D*_rot_, and the time step Δ*t*, the CMs also contain the moments 〈|*β*|^*n*^〉 of the tumble angle distribution *P*(|*β*|). We specify them for *E. coli* in Eq (20) and for *P. putida* in S2 Text.

*Even **n**:* The absolute value in the CM of Eq (6) needs special attention. Therefore, we first calculate the even CMs for our angular process Θ(*t*) governed by Eq (4):

$$m_{\Delta t}^{n}\left(\theta \right)\left[\Theta \right]=\left\langle \frac{{\left|d\Theta \right|}_{a}^{n}}{\Delta t}\;|\;\Theta \left(t\right)=\theta \right\rangle =\left\langle \frac{{\left(dN+dB\right)}^{n}}{\Delta t}\;|\;\Theta \left(t\right)=\theta \right\rangle \;\;\text{for}\;n\;\text{even}\;.$$ (7)

We apply the binomial formula, use that the increments *dB* and *dN* are uncorrelated, and obtain

mΔtn(θ)[Θ]=∑k=0k=nnk⟨dBk⟩(θ)⟨dNn-k⟩(θ)Δt. (8)

Eq (8) shows that we need to calculate the moments of the two stochastic processes separately. The odd moments of both increments vanish:

$$\left\langle dB^{k}\right\rangle =\left\langle dN^{k}\right\rangle =0\;\text{for}\;k\;\text{odd}$$ (9)

The even moments are included in the absolute moments, which we give for later use. For *dN* we assume that at most one shot occurs with rate λΔ*t* within the incremental time Δ*t*, and obtain

$${\left\langle |dN\right|}^{k}{\rangle \approx \lambda \left(\theta \right)\Delta t\langle |\beta |}^{k}\left(\theta \right)\rangle \;$$ (10)

where 〈|*β*|^*k*^〉 is the *k*-th moment of the distribution *P*(|*β*|) for the tumble angle. Note, that Δ*t* needs to be much smaller than the mean run time but larger than the mean tumble time. Thus, these two time scales should be separable, which is the case for the trajectories analyzed here for *P. putida* and *E. coli* [2, 38]. To calculate the moments of the Brownian process, we use the normal distribution of the increments with variance 2*D*_rot_Δ*t*,

$${\left\langle |dB\right|}^{k}\rangle ={\left[2D_{\text{rot}}\left(\theta \right)\Delta t\right]}^{k/2}\frac{2}{\sqrt{2\pi }}\int_{0}^{\pi /\sqrt{2D_{\text{rot}}\Delta t}}x^{k}\exp\left(-x^{2}/2\right)$$ (11)

For small *D*_rot_Δ*t* we can extend the upper limit of the integral to ∞ and obtain

$${\left\langle |dB\right|}^{k}\rangle ={\left[2D_{\text{rot}}\left(\theta \right)\Delta t\right]}^{k/2}\left(k-1\right)!!\left\{\begin{array}{cc} 1 & \text{for}\;\text{even}\;k\\ \sqrt{\frac{2}{\pi }} & \text{for}\;\text{odd}\;k \end{array}\right.\;,$$ (12)

where “!!” denotes the double factorial. Note that λ, *β* and *D*_rot_ may in general be functions of *θ*, for brevity we do not explicitly give the argument in the upcoming formulas.

We are now prepared to calculate the even CMs and start with *n* = 2:

$$m_{\Delta t}^{2}\left(\theta \right)\left[\Theta \right]=2D_{\text{rot}}+\lambda \left\langle \beta^{2}\right\rangle .$$ (13)

The first term on the right-hand side is derived from the mean-square displacement of Brownian motion, the second one comes from the shot noise, where we will specify *P*(|*β*|) and its moments in the following sections for each bacterium. The mixed binomial term vanishes according to Eq (9). For higher even moments we only consider terms up to linear order in the small square angular displacement *D*_rot_Δ*t*. Hence, only the second moment of Brownian motion appears in a mixed term:

$$m_{\Delta t}^{4}\left(\theta \right)\left[\Theta \right]\approx \lambda \left(\left\langle \beta^{4}\right\rangle +12D_{\text{rot}}\Delta t\left\langle \beta^{2}\right\rangle \right)$$ (14)

$$m_{\Delta t}^{6}\left(\theta \right)\left[\Theta \right]\approx \lambda \left(\left\langle \beta^{6}\right\rangle +30D_{\text{rot}}\Delta t\left\langle \beta^{4}\right\rangle \right)$$ (15)

$$m_{\Delta t}^{8}\left(\theta \right)\left[\Theta \right]\approx \lambda \left(\left\langle \beta^{8}\right\rangle +56D_{\text{rot}}\Delta t\left\langle \beta^{6}\right\rangle \right).$$ (16)

*Uneven **n**:* When applying Eqs (9), (10) and (12) for calculating the odd CMs, we assume the tumble angle to be larger than the Brownian angular step and refer to S1 Text for more details. We obtain for the first and third moment:

$$m_{\Delta t}^{1}\left(\theta \right)\left[\Theta \right]=\lambda \left\langle |\beta |\right\rangle +2\left(1-\lambda \Delta t\right)\sqrt{\frac{D_{\text{rot}}}{\pi \Delta t}}\;$$ (17)

$$m_{\Delta t}^{3}\left(\theta \right)\left[\Theta \right]={\lambda \langle |\beta |}^{3}\rangle +4\left(1-\lambda \Delta t\right)\sqrt{\frac{D_{\text{rot}}^{3}\Delta t}{\pi }}+6\lambda D_{\text{rot}}\Delta t\left\langle |\beta |\right\rangle \;.$$ (18)

Due to our definition of the CMs with absolute values, we obtain non-zero values for the odd moments as functions of the model parameters. We will compare the CMs from the analytical formula to moments calculated from experimental data and by a least-square fit determine the relevant parameters of bacterial motion. Note that the tumble rate λ enters linearly in each CM, while parameters related to the tumble angle *β* will, in general, be exponentiated up to the order of the moment. We will exploit this different scaling behavior of the CMs to identify a bias in the mean tumble angle during chemotaxis, which we will call angle bias in the following.

### Distribution of tumble angles for *E. coli*

To complete our model we have to specify the distribution of tumble angles |*β*|. For the *E. coli* bacterium, we are inspired by the seminal work of Berg and Brown [2] and choose a gamma distribution restricted to the domain [0, *π*] [40]:

$$P\left(|\beta |\right)=\gamma \left(\sigma ,k\right)=\frac{1}{\sigma^{k}\gamma_{inc}\left(k,\pi \right)}\;{\left|\beta \right|}^{k-1}\;e^{-|\beta |/\sigma }\;.$$ (19)

The lower, incomplete gamma function $\gamma_{inc}\left(k,x\right)=\int_{0}^{x}t^{k-1}\exp\left(-t\right)dt$ comes in when normalizing *P*(|*β*|) to one on the interval [0, *π*]. For *k* > 1 the gamma distribution has a maximum at (*k*−1)*σ*. Due to its particular scaling properties, each moment can be written in closed form:

$${\left\langle |\beta \right|}^{n}\rangle =\sigma^{n}\frac{\gamma_{inc}\left(k+n,\pi /\sigma \right)}{\gamma_{inc}\left(k,\pi /\sigma \right)}\;.$$ (20)

Realizations of the gamma distribution on the finite interval [0, *π*] are depicted in S3 Fig showing the wealth of different shapes, which can be achieved.

We analyze the bacterial trajectories with a time step Δ*t* = 0.5s, taking every tenth experimental data point, however, the conclusions we will draw in the following do not sensitively depend on this number. For *P. putida* and its tumble rate λ we explicitly demonstrate this in S4 Fig in the appendix. By shifting the starting point of the bacterial trajectories, we ultimately use all experimental data points in our analysis. In total, four parameters control the directional dynamics of *E. coli*: *D*_rot_, λ, *σ*, and *k*. We will infer them by analyzing bacterial trajectories with the help of the CMs defined in Eq (6).

### Calculating CMs from experimental trajectories

For each of the *N* experimental trajectories, we have a set of orientation angles Θ_*i*_(*t*) (*i* = 1…*N*) at discrete times *t*, which we use to determine CMs from the experimental data according to the following formula [30]:

$$m_{\Delta t}^{n}\left(\theta \right)=\frac{1}{Z_{K}}\sum_{i=1}^{N}\sum_{t}\frac{|\Theta_{i}\left(t+\Delta t\right)-\Theta_{i}{\left(t\right)|}_{a}^{n}}{\Delta t}\;\;K\left(\Theta_{i}\left(t\right)-\theta ,\Delta \theta \right)$$ (21)

with

$$Z_{K}=\sum_{i=1}^{N}\sum_{t}K\left(\Theta_{i}\left(t\right)-\theta ,\Delta \theta \right)\;.$$

Here, we average over all orientation angles of *N* trajectories, with a duration longer than 3 seconds. The Gaussian kernel $K\left(\Theta_{i}-\theta ,\Delta \theta \right)=\exp\left[-\frac{{\left(\Theta_{i}-\theta \right)}^{2}}{2\Delta \theta^{2}}\right]$ with Δ*θ* = 0.125*π* is used to condition the CMs on a defined angle *θ* in the presence of a chemical gradient. When we include all reorientation events to determine the CMs of the mean tumbling statistics, we simply set *K* = 1 in Eq (21), which corresponds to Δ*θ* → ∞.

To perform the parameter inference, we first use Eq (21) to determine a set of moments $m_{\Delta t}^{n}\left(\theta \right)$ from the experimental trajectories. For a given *θ* value, we then fit them with the analytical formulas for the CMs using the method of least squares, which we realize with a standard numerical optimization procedure from the python libary SciPy. This gives a set of parameters *D*_rot_, λ, *σ*, and *k*, which are in general functions of *θ*. We analyze trajectories recorded at different times *T* = 7, 12, 30, 45, 60, and 95 minutes after the start of the experiment. Typically, the tracks after 7 and 12 minutes, which we call “early” tracks, and the other “late” tracks are analyzed together. Errors of the inferred parameters are determined by a so-called bootstrap technique (see Ref. [41] and S4 Text).

### Experimental materials and methods

#### Cell culture

*E. coli* strain AW405 was streaked on 1.5% agar (AppliChem, Germany) containing Lysogeny broth (LB medium) (AppliChem, Germany) and grown at 37°C. A single-colony isolate was used to inoculate 10 ml of LB medium in a 100 ml flask and grown over night in shaking culture (300/min, 37°C). The stationary culture was diluted 1:100 into 10 ml of fresh LB medium and grown ≈ 3 h to an optical density at 600nm of OD_600_ ≈ 0.8 in mid-exponential phase. Bacteria were washed two times by centrifugation at 1000*g* for 10 min and carefully resuspended in 10 ml motility buffer (11.2 g/l K_2_HPO_4_, 4.8 g/l KH_2_PO_4_, 3.93 g/l NaCl, 0.029 g/l EDTA and 0.5 g/l glucose; pH 7.0). Cells were diluted further to an OD_600_ of 0.05 before filling them into the chemotaxis device.

#### Chemotaxis assay and imaging

**Fig 3:**
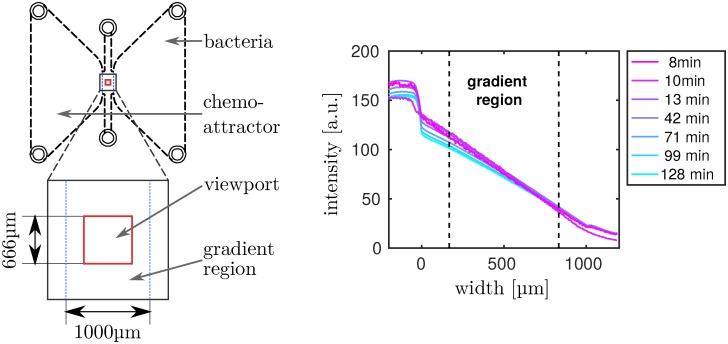
Experimental setup. **Left:** Layout of the chemotaxis chamber. Attractant reservoir is on the left, cell reservoir on the right. The central gradient region is marked in blue, its height is 70 μm, much less than the height of the reservoir chambers. Because of the significantly larger volume in the chambers, a linear gradient establishes after filling and is maintained for several hours. Marked in red is the field of view imaged by the microscope. **Right:** Temporal evolution of the chemical gradient profile after filling the channel, measured from the spatial profile of fluorescein. Since fluorescein has about twice the molecular weight of *α*-methyl-aspartate and thus a larger diffusion coefficient, we assume that the gradient evolution measured for fluorescein is similar or slightly slower than the gradient of the chemoattractant.

We used a μ-Slide Chemotaxis 3D (ibidi, Martinsried, Germany) to generate stable linear gradients of the chemoattractant *α*-methyl-aspartate (Sigma-Aldrich, USA). First, the gradient region was filled with motility buffer, then the cell suspension was filled into the right reservoir of the channel. Lastly, the left reservoir was filled with motility buffer containing 0.5 *α*-methyl-aspartate. Imaging was done using an IX71 inverted microscope with a 20× UPLFLN-PH objective (both Olympus, Germany) in phase contrast mode with an attached Orca Flash 4.0 CMOS camera (Hamamatsu Photonics, Japan). All data was aquired at 20. Video sequences of 2 minutes each were recorded at 7, 12, 30, 45, 60 and 95 minutes after filling the channel. By recording control datasets with 1 μM fluorescein added to the chemoattractant reservoir, we confirmed that the gradient was already established at the time of recording. Using confocal laser scanning microscopy on a channel filled only with buffer and buffer with fluorescein, we estimated the concentration profile of the chemoattractant at different times after filling. As shown in Fig 3 on the right, the gradient is approximately linear already at 7 minutes after filling, and remains almost stationary for the duration of the experiment. The field of view and focal plane were set in the center of the gradient region and about 35 μm from top and bottom of the 70 μm high channel (see Fig 3 left). Because the depth of focus for this setup was about 5 μm, our recordings represent quasi-2D slices within the channel volume.

#### Image processing and cell tracking

Cells were observed using a 20x phase contrast objective from Olympus. Together with the Hamamatsu Orca Flash camera, this setup gives the following focal volume: 661*μm* × 661*μm* × 2.9*μm*. Image sequences were exported from the camera manufacturer’s native data format to BigTIFF sequences and further processed using a custom program written in Matlab (version R2014a, The MathWorks, USA). For each image stack the pixelwise average projection was calculated, which was then used for background correction. Dividing each frame by the background image yielded an image, corrected for shading effects and which was free of non-moving objects. The resulting background corrected image stack was segmented using a Matlab version of the Maxentropy thresholding algorithm by Kapur et al. [42]. To minimize noise introduced by the segmentation, the threshold was computed for each image separately and the median of these values was used to segment the whole stack. Small specks present after segmentation were removed by morphological opening and closing with a disk of equivalent radius of 0.3 μm. Positions of cells were determined by computing the centroid of each connected component in the binary image. Afterwards, only objects with areas between 1 μm^2^ to 15.6 μm^2^ were considered as single cells and used for further analysis. Finally, cell tracking was performed using a Matlab version of the particle tracking algorithm by Crocker and Grier [43].

#### Filtering

Cells tend to tumble at the beginning and end of each trajectory, because this is the most common way to enter the focal plane. If they swim at an angle to the plane, they only appear very briefly and are not tracked. Thus we disregard the first and last 0.5 s of each recorded track in order to avoid any biasing in the tumble rates. Because run-and-tumble detection is not feasible with very wobbly tracks, the dataset was filtered based on several criteria. Trajectories with a duration longer than 10 s or with a total displacement below 10 μm were discarded. Additionally, we removed the 20% of tracks with the highest median curvature [27]. This curvature filter effectively removes tracks, where the cells tumble for very long times.

#### Run-and-tumble recognition

For the heuristic run-and-tumble recognition we subsampled the original track data by using only every third data point yielding to an effective data rate of 6.6 Hz. Furthermore, we smoothed the tracks by applying a 5-point, second-order Savitzky–Golay filter [44]. The smoothed tracks were used to compute frame-wise speed $v=\frac{\Delta s}{\Delta t}$, direction of propagation *θ*, and turning rate $\omega =\frac{\Delta \theta }{\Delta t}$. To identify tumble events, we used the algorithm from Theves et al. [38] with parameters adjusted to give reasonable results for our data sets. In short, this algorithm determines tumble events by evaluating local extrema in time series of speed and turn rate. A tumble event is identified if the speed minimum is sufficiently deep or the maximum in the turn rate sufficiently large. More details of the algorithm are described in the supporting information (see S5 Text).

### Software

The software, where we implemented the method of conditional moments, is freely available as python code via https://github.com/OliverPohl/Conditional-Moment-Method. It includes the calculation of the moments from experimental trajectories, the inference of the parameters by a least-square fit, and the tumble recognizer. We make use of the python packages “numpy”, “scipy”, and, in particular, of “scipy optimize”.

## Results

In this section, we analyze experimental trajectories of *E. coli* cells recorded in a linear gradient of chemoattractant and use the method of conditional moments to infer the tumbling statistics of this organism in two cases. First, we determine the mean tumbling statistics irrespective of the orientation of the swimmers relative to the chemical gradient. Second, we explore chemotaxis of *E. coli* by analyzing its tumbling statistics conditioned on its orientation. In the first case, we also demonstrate a novel tumble recognizer based on our method of conditional moments. Finally, we analyze trajectories of the bacterium *P. putida* and discuss its chemotactic strategy.

### Overall tumbling statistics of *E. coli*

In order to test our method of conditional moments against a commonly used heuristic tumble recognizer, we focus on the overall tumbling statistics of *E. coli* irrespective of the swimming direction of cells relative to the chemical gradient. Thus, we set *K* = 1 in Eq (21) and disregard the condition *θ*.

We consider the heuristic tumble recognizer as described in Refs. [25] and [38] (see also the materials section and S5 Text). We use it to select a set of trajectories *S*_1_ with at least one recognized tumble in all the late data sets at 30, 45, 60, and 95 min. With our inference method we obtain a mean tumble rate λ_*i*_ = 0.39 ± 0.03 s^−1^, which nicely agrees with λ_*c*_ = 0.39 ± 0.01 s^−1^ determined with the heuristic tumble recognizer. Previously reported values range from 0.42 s^−1^ [20] to 2.86 s^−1^ [27] in two-dimensional setups and give 1.12 s^−1^ in three dimensions [2]. Because of our experimental setup, only cells within a narrow focal zone (about 3 μm thick compared to 8 μm in [20, 27]) are recorded. Hence, the most frequent way for cells to enter or to leave the narrow focal plane is by tumbling. However, these two tumble events per track do not enter our analysis because their tumble angles cannot be recorded. Upon adding them, the average tumble rate increases to λ = 0.92 s^−1^.

**Fig 4:**
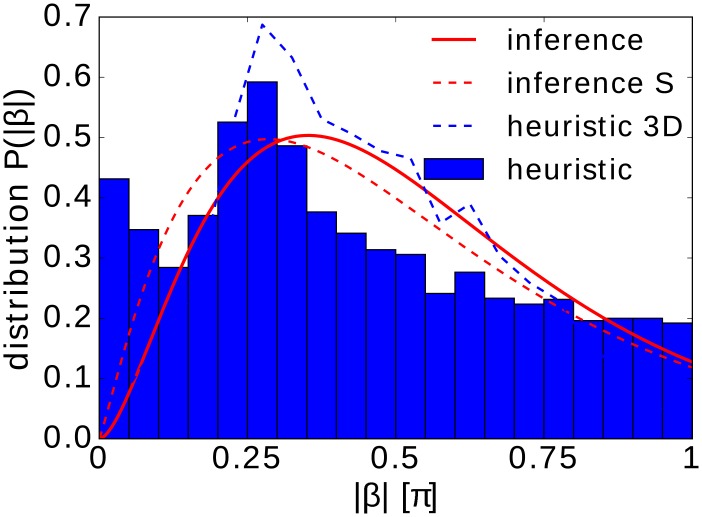
Distribution of tumble angles, *P*(|*β*|). It is determined from experiments by the heuristic tumble recognizer (bar graph) and by the inference method with the gamma function *γ*(*σ*, *k*) as an ansatz (solid red line). All recorded trajectories at 30, 45, 60, and 95 min with at least one tumble are used. The mean tumble angle and the standard deviation are 〈|*β*|〉 = 0.42*π* = 76.0°, Δ|*β*| = 0.27*π* = 48.7° (heuristic tumble recognizer) and 〈|*β*|〉 = 0.47*π* = 85.4°, Δ|*β*| = 0.23*π* = 41.8° (inference method). The inferred parameters of *γ*(*σ*, *k*) are *σ* = 0.64 and *k* = 2.73. The red dashed line refers to the inferred gamma distribution (*σ* = 0.78 and *k* = 2.15), when the original data is smoothed. The blue dashed line refers to the histogram values multiplied by sin(|*β*|) and then normalized to one, thus representing the tumble angle distribution in three dimensions.

Fig 4 compares the distribution of tumble angles determined with the tumble recognizer and the method of conditional moments. They share common features: a maximum at 50° or 63°, a skewed shape, and a considerable amount of large tumble angles. The main difference of both distributions in Fig 4 is the absence of small tumble angles in the inferred distribution, for which we assume a gamma function. Thus, possible small tumble angles do not enter in the inferred tumbling statistics, they are rather classified as Brownian noise. To include small tumble angles in the inference method, one would need an alternative ansatz function for the distribution *P*(|*β*|). Finally, unlike classical heuristic and other tumble recognizers, we infer *D*_rot_ from the available data rather than using a fixed value in our analysis. We find *D*_rot_ ≈ 0.06 ± 0.01 s^−1^ confirming the literature value of *D*_rot_ ≈ 0.062 s^−1^ [45].

We add two remarks. First, when we use smoothed trajectories in our inference method as the heuristic tumble recognizer does, we also obtain a maximum tumble angle of ca. 50° (see dashed red line in Fig 4). The reason is that sharp edges in the bacterial trajectories are smoothed. However, we prefer to perform the inference method with the raw data without any additional parameters to be chosen.

Second, the tumble events are recorded when the three-dimensional trajectories run in a specific plane. All planes defined by the bacterial path before and after a tumble event are equivalent. So, to obtain the distribution of tumble angles for the three-dimensional trajectories, we just have to multiply *P*(|*β*|) with sin|*β*| from the differential solid angle *dΩ*. Indeed, the resulting distribution (see dashed blue line in Fig 4) compares well to the one reported in Ref. [2]. In particular, it becomes zero at |*β*| = 0 and *π* and it has a peak reported at about 50°.

### Defining a novel tumble recognizer

**Fig 5:**
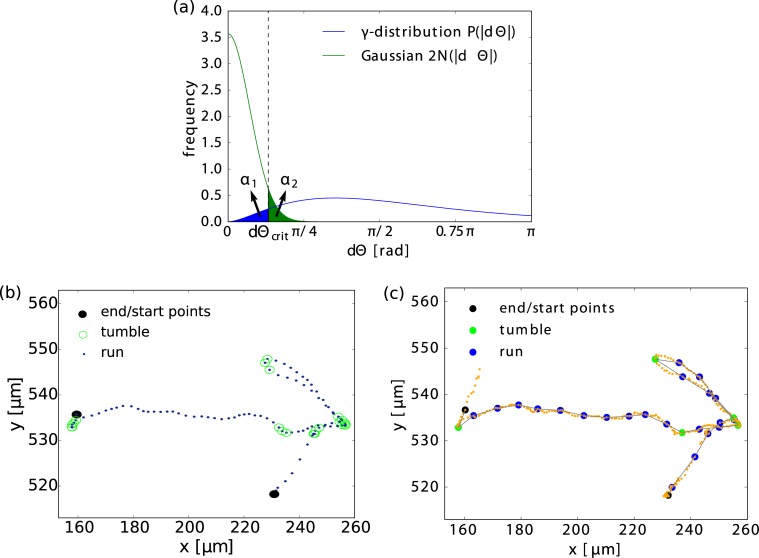
Definition and analysis of systematic tumble recognizer. (a) Distributions for tumble angles, *P*(|*d*Θ|) (blue line), and for Brownian displacements, 2N(|dΘ|) (green line), as inferred from experimental data. The shaded blue area corresponds to the type-I error *α*_1_, while the green area refers to the type-II error *α*_2_. The dashed line marks the threshold *d*Θ_crit_. (b) Smoothed sample trajectory with tumbles marked as green circles. They are obtained by a heuristic tumble recognizer (see S5 Text). Endpoints are not classified and therefore black. (c) Rational tumble recognition on the basis of a hypothesis test with the likelihood ratio using the inferred distributions *P*(|*β*|) and N(dΘ). Faint orange points are the original trajectory points, colored fat points are trajectory points with time gap Δ*t* = 0.5.

Averaging over all late experimental trajectories, we have trained our model by adjusting its parameters. In particular, we know the probability distribution *P*(|*β*|) of the tumble angle *β* as well as the probability density for thermal angular displacements, N(dΘ), which is a normal distribution with mean 0 and variance 2*D*_rot_Δ*t*. As can be seen in Fig 5(a), both distributions do have considerable overlap. This raises the question if a given angular displacement *d*Θ from the overlap region is due to Brownian diffusion or due to tumbling. Hence, the task to identify a tumble event based purely on the angular displacements contains some intrinsic uncertainty. Also any heuristic tumble recognizer contains such an uncertainty, since threshold parameters have to be fixed (see S5 Text). Typically, a threshold value is used to define a minimal angular velocity associated with a tumble [2]. Recently, the reduced speed of the bacteria during tumbling was introduced as an additional criterion in heuristic tumble recognizers [25, 38]. It allows for the detection of tumble events with very small *d*Θ. However, the speed statistics is noisy and one needs to introduce two additional threshold parameter, which in turn leads to further uncertainties in identifying tumble events (see S3 Text).

In the following, we define a novel tumble recognizer, which we call systematic. It makes the probabilistic character of tumbling recognition explicit by quantifying the uncertainty in recognizing a tumble event. To this end, we rely on the framework of statistical hypothesis testing [46]. Intuitively, if for a given reorientation angle *d*Θ the tumble probability density *P*(|*d*Θ)| is larger than 2N(dΘ), we would call the event a tumble. Accordingly, we introduce the *likelihood ratio*

$$R\left(d\Theta \right)≔\frac{2N(d\Theta )}{P(|d\Theta |)}\;.$$ (22)

If *R* is small, we accept the hypothesis H: “*d*Θ belongs to a tumble”. If *R* is large, we reject it and claim that *d*Θ is of thermal origin. Thus we need to introduce a threshold *r*_crit_ such that we accept our hypothesis whenever *R* < *r*_crit_(*α*_1_). Here, the threshold value *r*_crit_ depends on the confidence level *α*_1_, which is the so-called *type-I error* or the probability that we miss a tumble. When we introduce a threshold value *d*Θ_crit_ by the implicit equation *R*(*d*Θ_crit_) = *r*_crit_ and assign all angular displacements smaller than *d*Θ_crit_ to Brownian motion, we are able to quantify the confidence level as [see Fig 5(a)]

$$\alpha_{1}=\int_{0}^{d\Theta_{\text{crit}}}P\left(|d\Theta |\right)d\left(d\Theta \right)\;.$$ (23)

In our case, we choose *α*_1_ = 0.05, determine *d*Θ_crit_ = 24° from Eq (23), and ultimately *r*_crit_(*α*_1_) = 2.3 from *r*_crit_ = *R*(*d*Θ_crit_). Once given *α*_1_ and *d*Θ_crit_, we can calculate the *type-II error* *α*_2_ or the probability that by mistake we recognize a tumble: $\alpha_{2}=\int_{d\Theta_{\text{crit}}}^{\pi }N\left(d\Theta \right)d\left(d\Theta \right)\approx 0.06$.

This hypothesis test based on the likelihood ratio *R* is also called Neyman-Pearson test [47]. It has the property of optimality in the sense that, given the two distributions and the confidence level *α*_1_, there is no other test with smaller *α*_2_.

We apply this test to a representative trajectory plotted in Fig 5(c) and compare it to the track divided in tumbles and runs by the heuristic tumble recognizer [Fig 5(b)]. We see that most of the recognized tumbles are identical. Only one tumble is identified by the heuristic tumble recognizer, which is marked as run with the systematic tumble recognizer. The heuristic recognizer detects a faint speed minimum at this spot, whereas the angular change is insufficient for the systematic recognizer to tag the event as a tumble. We checked that the systematic tumble recognizer identifies 85% of tumbles and runs detected by the heuristic recognizer.

Hence, with our proposed tumble recognizer the uncertainty in tumble recognition is quantified by the type I and II errors. There are no unknown parameters, which have to be set a priori besides the sampling rate. To improve the performance further, one might consider time series in the swimming speed in addition to the orientation angle.

### Chemotaxis of *E. coli*

Now, we infer the specifics of the tumbling behavior of *E. coli* while performing chemotaxis. Therefore, we condition on the orientation angle *θ* by setting the angular width in Eq (21) to ΔΘ = 0.125*π*. However, due to Eq (5) we expect the tumble rate λ(*t*) at time *t*, to depend on the whole past of the trajectory. So, why is conditioning on the orientation angle *θ* sufficient? The response function *R* is typically peaked at times close to zero meaning that the response to the very recent past is weighted strongest [24]. Since the bacterium moves persistently between two tumbles, we expect a strong dependence on the orientation *θ* just before tumbling. We will make this insight more quantitative in Eq (24) below. Indeed, in Eq (6) one could also condition on the whole bacterial trajectory, which will then reveal details of the response function. This will be discussed elsewhere. To determine the conditional moments, we consider all trajectories, which are longer than 3 seconds irrespective of whether they contain tumble events or not. This, of course, leads to a lower tumble rate than for the trajectory set *S*_1_ analyzed above.

**Fig 6:**
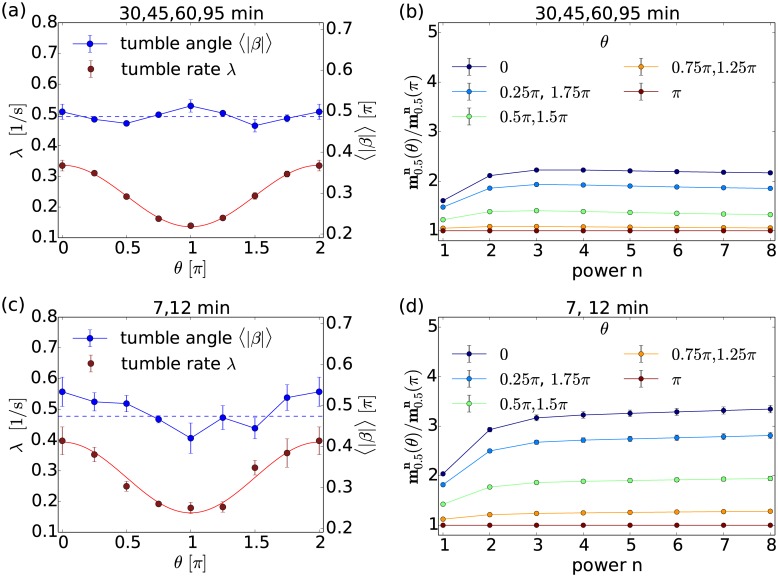
Tumbling statistics with chemotaxis. (a), (c) The mean tumble rate λ (red) and the mean tumble angle 〈|*β*|〉 (blue) plotted versus the orientation angle *θ* of the bacterium prior to tumbling for (a) the late tracks at *T* = 30, 45, 60, and 95 min and for (c) the early tracks at *T* = 7 and 12 min. The tumble rate is fitted by a cosine function. The dashed blue line marks 〈|*β*|〉(*θ*) averaged over all directions. (b), (d) Ratios of CMs, $m_{\Delta t}^{n}\left(\theta \right)/m_{\Delta t}^{n}\left(\pi \right)$, plotted versus power *n* for different orientation angles *θ* for (b) the late tracks and for (d) the early tracks.

In the left column of Fig 6 we plot the inferred tumble rate λ(*θ*) (red curve) and the mean tumble angle 〈|*β*|〉(*θ*) (blue curve) at different times *T* after the start of the experiment. To determine 〈|*β*|〉(*θ*), we use the inferred parameters *k*(*θ*) and *σ*(*θ*) in Eq (20).

#### Tumble rate

We find that at early and late times *T*, λ(*θ*) is essentially symmetric about its minimum *θ* = *π*, where the bacterium moves along the chemical gradient (see Fig 2). Indeed, the data is well fitted by a shifted cosine function, λ_fit_(*θ*) = *a*_1_ + *a*_2_ cos(*θ*), with two fit parameters *a*_1_ and *a*_2_. This can be rationalized by means of Eq (5). Since the chemical gradient is directed along the negative *x*-direction, we can approximate the integral in Eq (5) by the following sum representing the runs of the bacterium between two tumbles:

$$\lambda \left(t\right)=\lambda_{\text{equ}}+\cos\left(\theta \right)Kv_{0}\int_{t_{0}}^{t}R\left(t-t^{\prime }\right)tdt^{\prime }+\sum_{i=0}^{n}\int_{t_{i+1}}^{t_{i}}\cdots \;,$$ (24)

where *K* is the magnitude of the constant chemical gradient. In the right-hand side of Eq (24) we neglect the sum of the third term resulting from runs before the last tumble. We assume that this sum vanishes when averaging over many trajectories. Hence, comparing our fit function with Eq (24), we recognize that *a*_1_ approximates the mean tumble rate λ_equ_ and *a*_2_ ∝ *Kv*_0_ is a measure for the strength of the chemotactic response. The plots in Fig 6(a) and 6(c) reveal that for the late and early trajectories λ_equ_ and chemotactic strengths are very similar: *a*_1_ = 0.28 s^−1^ and *a*_2_ = 0.11 s^−1^ for the early trajectories, and *a*_1_ = 0.24 and *a*_2_ = 0.10 for the late tracks. Therefore, our method reproduces and quantifies the classical chemotaxis strategy, i.e., adaption of λ according to the gradient direction.

#### Mean tumble angle

Surprisingly, early and late trajectories behave differently for the mean tumble angle 〈|*β*|〉(*θ*). While in the late trajectories it is roughly constant in *θ* [blue curve in Fig 6(a)], a minimum around *θ* = *π* is recognizable in the early tracks [Fig 6(c)]. This indicates that at early times of the experiment tumble angles are biased towards smaller values when *E. coli* moves along the chemical gradient. Such an angle bias was also reported in Ref. [26]

To support our findings, we determine the CMs from the experimental data and plot ratios of the form $m_{\Delta t}^{n}\left(\theta \right)/m_{\Delta t}^{n}\left(\pi \right)$ versus order *n* in Fig 6(b) and 6(d). From Eqs (13)–(18) we find that for *n* > 3 the moments are mainly determined by the leading term λ〈|*β*|^*n*^〉 since *D*_rot_Δ*t* ≪ 1 and thus

$$\frac{m_{\Delta t}^{n}\left(\theta \right)}{m_{\Delta t}^{n}\left(\pi \right)}\approx \frac{\lambda (\theta )}{\lambda (\pi )}\frac{\left\langle |\beta {|}^{n}\left(\theta \right)\right\rangle }{\left\langle |\beta {|}^{n}\left(\pi \right)\right\rangle }\;.$$ (25)

Since the tumble rate λ(*θ*) is smallest along the chemical gradient (*θ* = *π*), we expect this ratio of CMs to increase with growing |*θ* − *π*| for each *n*. This is confirmed by the graphs in in Fig 6(b) and 6(d) for a fixed *n*. More importantly, the ratio provides a mean to clearly distinguish between classical chemotaxis and a strategy with angle bias: If the ratio in Eq (25) increases with growing *n*, we must have 〈|*β*|(*π*)〉 < 〈|*β*|(*θ*)〉 for *θ* ≠ *π* and hence an angle bias. In contrast, if the ratios for different *θ* converge towards constant values at larger *n*, we confirm classical chemotaxis with 〈|*β*|(*π*)〉 = 〈|*β*|(*θ*)〉. Therefore, inspecting the ratio of CMs provides a method to distinguish chemotactic strategies directly from the experimental data without any fitting procedure involved. For the late trajectories we find the expected convergence towards nearly constant values at roughly λ(*θ*)/λ(*π*) [see Fig 6(b)]. However, for the early trajectories the ratios in Fig 6(d) show a small but clearly recognizable increase with *n*, which hints to an angle bias. This result led us to perform a more careful analysis of the early trajectories at *T* = 7 and 12 min.

At early times the bacterial population is divided into chemotactically efficient and less efficient swimmers along the *x*-direction of the channel. To demonstrate this, we divide the field of view of our experimental setting in Fig 3 in a right, middle, and left part (all three of the same width 222*μ*m along the gradient direction), and condition the CMs also on the location in either of these parts. A bacterium moves with a chemotactic drift velocity along the gradient. For the chemical gradient employed in our experiments a typical value for *E. coli* is 0.9*μ*m/s [2]. Thus, to reach all locations in the left part of the field of view, an average bacterium needs ca. Δ*t* = Δ*s*/*v* = 800*μ*m/(0.9*μ*m/s) ≈ 900 s or 15 min. Given the additional fact that at the initiation of the experiment the chemotactic gradient has not been established yet, we conclude that only chemotactically fast bacteria can reach the left part of the field of view and be recorded 7 or 12 minutes after the start of the experiment. At later times also chemotactically slower bacteria reach the left part and the bacterial population is well mixed.

**Fig 7:**
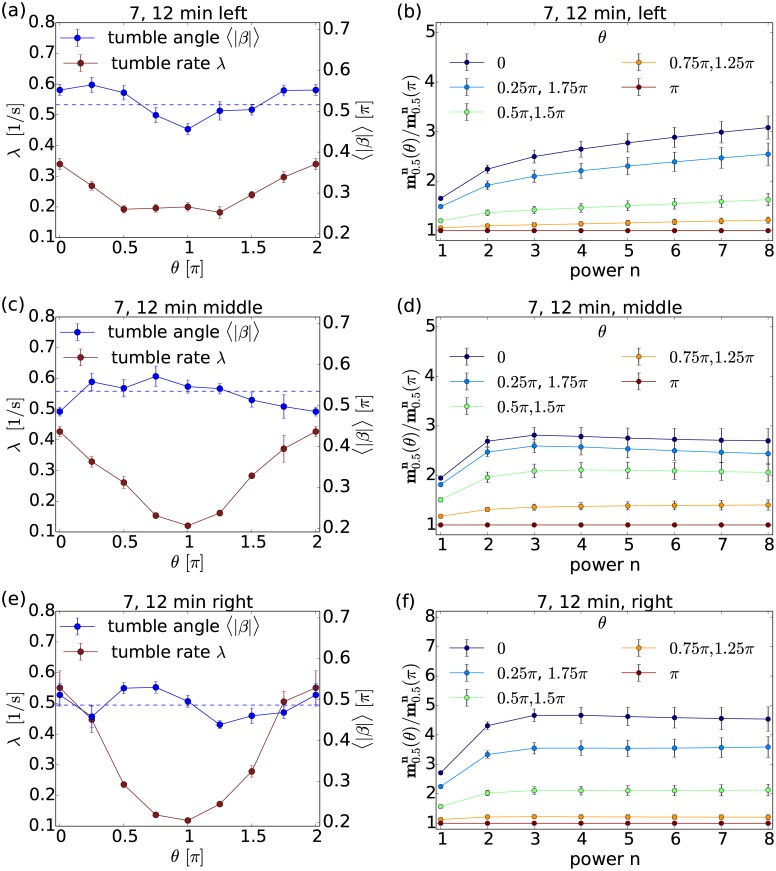
Early bacterial tracks analyzed separately in different parts of the channel. Left column: The mean tumble rate λ (red) and the mean tumble angle 〈|*β*|〉 (blue) plotted versus the orientation angle *θ* prior to tumbling for (a) the left, (c) the middle, and (e) the right part. The blue dashed line marks 〈|*β*|〉(*θ*) averaged over all directions. Right column: Ratios of CMs, $m_{\Delta t}^{n}\left(\theta \right)/m_{\Delta t}^{n}\left(\pi \right)$, plotted versus power *n* for different orientation angles *θ* for (b) the left, (d) the middle, and (f) the right part.

In Fig 7 we demonstrate that the chemotactically efficient bacteria apply an angle bias as additional chemotaxis strategy. We plot the mean tumble angle and the ratio of CMs, which are also conditioned on the left [(a), (b)], middle [(c), (d)], and right [(e), (f)] part of the channel. Fig 7(a) and 7(b) show strong indication for an angle bias in the left part of the channel. The ratios of CMs clearly increase with *n*, as discussed before, whereas for bacteria in the middle and right part the curves are essentially constant or even fall [see Fig 7(d) and 7(f)]. This cannot be the outcome of statistical fluctuations. Since we measure the tumble angles locally, it is very unlikely that all the bacteria show their incidentally biased tumble angles in the left part. In conclusion, we give evidence that some *E. coli* cells in our experiments apply an angle bias to swim along chemical gradients more efficiently. In Ref. [48] a mean tumble angle anticorrelated with the speed of bacteria was measured in the absence of a chemical gradient. This is fundamentally different to our our observation, where the mean tumble angle is correlated with the bacterial orientation relative to the chemical gradient before tumbling. In Ref. [49] it is proposed that bacteria adapt tumble times to their moving direction and thereby introduce an angle bias.

The tumble rate, on the other hand, shows a stronger dependence on *θ* for bacteria on the right compared to the left side of the channel [red curves of Fig 7(a), 7(c) and 7(e)]. This effect is also observed for all later stages of the experiment (see, for example, graphs for *T* = 60 min. and *T* = 30, 45 min. in S1 Fig of the supporting information). So, in contrast to the angle bias it is not special for the early data acquisitions. In Ref. [50] logarithmic sensing of the chemical gradient has been reported, meaning that the chemotactic response scales with ∇*c*/*c* = ∇(log*c*). This could explain the stronger chemotactic response of the tumble rate λ at the right side of the channel, where *c* is small.

### Chemotaxis of *P. putida*

In this section, we apply the inference method based on CMs to the bacterium *P. putida*, which performs a notably different random walk than *E. coli* [3, 38]. During tumbling it typically reverses its direction of motion. Our data set consists of trajectories taken more than one hour after the start of the experiment, which we analyze in the following. Furthermore, we set Δ*t* = 0.3 s, which is smaller than in the case of *E. coli*, because the tumbles are typically shorter. However, as before, the exact value of Δ*t* does not alter the results qualitatively (see S4 Fig). Last, we need to specify the distribution of tumble angles, *P*_put_(|*β*|). In Ref. [38] such a distribution was determined in experiments. It shows one strong peak at 180° (reversal) and a small one at 0°, which corresponds to “stopping” events without any reorientation. These events cannot be identified without inspecting the bacterium’s speed. Since our inference method only considers the orientation angle Θ, we are not able to detect it. Thus, concentrating on the strong peak at 180°, we choose for the tumble angle distribution

$$P_{\text{put}}\left(|\beta |\right)=\frac{1}{N}\left(e^{-(\pi -|\beta |)/\Delta \beta }+C\right),$$ (26)

where N is the normalization constant and *C* is a typically small offset to fit the occurrence of small tumble angles. In S2 Text we list the moments 〈|*β*|^*n*^〉 of the tumble angle distribution needed to calculate the CMs. By fitting them to the experimentally determined CMs, we infer the parameters *D*_rot_, λ, Δ*β*, and *C*.

**Fig 8:**
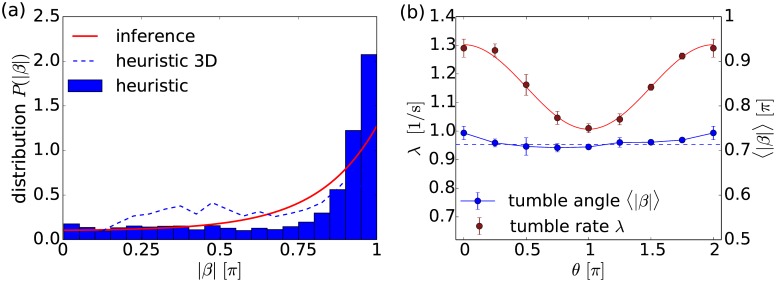
Tumbling statistics of *P. putida*. (a) Distribution of tumble angles, *P*(|*β*|), determined by the heuristic tumble recognizer (bar graph) and by the inference method (red line). The blue dashed line refers to the histogram values multiplied by sin(|*β*|) thereby representing the tumble angle distribution in three dimensions. The mean tumble angle and the standard deviation are 〈|*β*|〉 = 0.75*π* = 135°, Δ|*β*| = 0.29*π* = 52.2° (heuristic tumble recognizer) and 〈|*β*|〉 = 0.72*π* = 130°, Δ|*β*| = 0.26*π* = 46.8° (inference method). (b) The mean tumble rate λ (red) and the mean tumble angle 〈|*β*|〉 (blue) plotted versus *θ*. The tumble rate is fitted by a cosine function.

First, we determine these parameters without conditioning on a specific value *θ* for the orientation angle, in order to infer the tumble angle distribution plotted as a red line in Fig 8(a). It captures the trend of the distribution determined with the heuristic tumble recognizer. In particular, the peak at *β* = *π* is clearly recovered and the mean tumble angles of both distributions nicely agree [see caption of Fig 8(a)]. Furthermore, when plotting the normalized distribution ∝ sin(|*β*|)*P*(|*β*|) to capture the three-dimensional tumble angle distribution (dashed blue line), one recovers a bimodal shape as reported in Ref. [51].

When conditioning on the orientation angle *θ* prior to a tumbling event, we first note that the mean tumble angle 〈|*β*|〉 = 0.72*π* ± 0.03*π* is approximately constant [see Fig 8(b)]. This is also confirmed by the ratios of CMs, which clearly converge to constant values (see S2 Fig). The mean tumble rate λ averaged over *θ* is significantly larger for *P. putida* compared to *E. coli*, ∫λ*dθ*/2*π* ≈ 1.1 s^−1^. Finally, we see that *P. putida* biases its tumble rate similar to *E. coli* meaning that it applies a classical chemotaxis strategy. The tumble rate is again well approximated by λ_fit_(*θ*) = *a*_1_ + *a*_2_ cos(*θ*) with *a*_1_ = 1.15 s^−1^ and *a*_2_ = 0.15 s^−1^.

## Discussion

In this article we presented a novel method to infer the relevant parameters of bacterial motion from experimental trajectories including the tumbling events. To this end, we defined a basic stochastic model for the run-and-tumble random walk, where shot noise initiates a tumble event in the orientation angle. We analytically calculated conditional moments within the model and matched them to the moments determined from recorded data of experimental trajectories. Applying the new technique to the bacteria *E. coli* and *P. putida*, we were able to infer the respective mean tumble rates, the rotational diffusion constants, and the distributions of tumble angles. Based on the inferred parameters we also introduced a new tumble recognizer using the framework of statistical hypothesis testing. In contrast to heuristic tumble recognizing procedures, there is only one parameter necessary, the confidence level, which quantifies the uncertainty or the error in recognizing all tumble events. Thus, the new tumble recognizer makes explicit the uncertainty in tumble recognition, which is inherent to any recognizer.

Conditioning the moments on the orientation angle, we explored and quantified the chemotactic strategy of the two bacterial species. Although *P. putida* performs a different random walk than *E. coli*, frequently reversing its direction of motion by 180°, we showed that it applies the classical chemotaxis strategy of bacteria. For *E. coli* we could detect some swimmers, that reduce their mean tumble angle significantly when moving along the chemical gradient. This confirms the angle bias reported in Ref. [26], which was obtained by averaging over all individuals of a *E. coli* cell population. Here, we gave evidence that only a part of the cell population exhibits this angle bias. Our findings are supported by a scaling analysis of appropriate CM ratios, which are directly calculated from experimental data.

We have tested our novel method of conditional moments for the well known random walk of *E. coli* and also discovered a new feature in its chemotactic strategy. Our method is formulated in general terms and can be applied to different bacterial random walks with a run-and-tumble strategy as demonstrated for *P. putida*. We mention other possible applications. For instance, magnetotactic bacteria [13, 14] experience a torque, which aligns their directions of motion with a magnetic field. Such an angular drift is easily included in the stochastic equation for the orientation angle and thereby well captured in the context of our inference method by means of the first conditional moment. Marine bacteria possess a bimodal distribution of tumble angles with two maxima as measured in Ref. [52]. In order to quantify this bimodality within our inference method, one first needs to specify a sufficiently general ansatz for the tumble distribution as input in the model. Then, a bimodality coefficient based on the third and fourth moments can be introduced and compared to experimental data [53].

Since variations in speed and orientation are strongly coupled to each other during a tumble event (different than discussed in Ref. [54]), we neglected speed fluctuations in a first approach. However, our inference method can be extended by including temporal variations in speed, which for *P. putida* should model the alternating speeds of propagation and might also permit to identify so-called “stopping events” [38]. Furthermore, such a modeling could also increase the precision of our tumble recognizer. For example, for *E. coli* one can model the time-dependent speed *v*(*t*) in Eq (1) as an Ornstein-Uhlenbeck process: $\dot{v}=r\left[v_{0}-v\left(t\right)\right]-\tilde{q}\left(t\right)+\mu$. The shot-noise process $\tilde{q}$ has spikes located at the same time points as the shot noise *q* for the tumble events and some suitable amplitude. The mean speed of all bacteria is represented by *v*_0_ and *μ* is Brownian white noise. The moments are readily calculated and one also obtains an estimate for the duration of a real tumble event, the tumble time *τ* ≈ *r*^−1^. Such an approach will be presented elsewhere. It will be more elaborate for *P. putida* due to the features mentioned before and reported in Ref. [38].

Our inference method based on conditional moments revealed a bias of tumble angles for some of *E. coli* cells during chemotaxis. However, the mechanism behind this effect is unknown. Ref. [55] reports a coordinated reversal of flagellar motors on a single *E. coli* cell initiating tumbling. The authors speculate that such correlations could lead to the observed angle bias. Indeed, the positions of attachment as well as the number of flagella significantly vary between different bacterial species [56] and also between individuals of one single species [57]. Possibly, this phenotypic diversity is the origin of the non-uniform chemotactic strategy we find. The method elaborated throughout this article provides a flexible framework to uncover such strategies hidden in the statistics of bacterial tumbling.

## Supporting Information

S1 Text Calculating odd CMs. (PDF)

S2 Text Moments of the tumble angle distribution of *P. putida*. (PDF)

S3 Text Trajectory analysis. In this appendix, two sample trajectories are presented and used to discuss the difference between heuristic and systematic tumble recognizer.
(PDF)

S4 Text Bootstrap method. The bootstrap method is presented, which allows to determine the variances of the inferred parameters from one set of trajectories.
(PDF)

S5 Text Heuristic tumble recognizer. Details on the heuristic tumble recognizer are provided. In particular, we explain the threshold parameters, needed to determine tumble events.
(PDF)

S6 Text Non-exponential run-time distributions. We show that our approach is valid for run-time distributions, which have a finite first moment.
(PDF)

S1 Fig Position analysis of late trajectories. Tumble rate, mean tumble angle, and CM ratios for the late trajectories of *E. coli* in the left, middle, and right part of the channel are provided.
(PDF)

S2 Fig Ratios of CMs for *P. putida*. (PDF)

S3 Fig Various gamma distributions for different parameters. (PDF)

S4 Fig Tumble rate λ of *P. putida* determined for different time steps Δ*t*. (PDF)
