# Supplementary material for: Inferring the Chemotactic Strategy of P. putida and E. coli Using Modified Kramers-Moyal Coefficients
Source: PLoS Comput Biol. 2017 Jan 23;13(1):e1005329. doi: 10.1371/journal.pcbi.1005329 (PMC5293273; doi:10.1371/journal.pcbi.1005329)
Supplement: S1 Text — (PDF) [file pcbi.1005329.s001.pdf]

S1. Calculating odd CMs

Calculating the CMs for odd power n is not straightforward because they are defined with absolute values as base. Here, we explain in detail the calculations of the first and third moments.

Using the identity $|a+b| = 2\max(a,-b) - a + b$, we obtain for the first moment:

$$\begin{aligned} m^1_{\Delta t}(\theta)[\Theta] &= \langle |dN_{\Delta t} + dB_{\Delta t}|/\Delta t \mid \Theta(t) = \theta \rangle \\ &= \langle 2\max(dB, -dN) - dB + dN \rangle/\Delta t. \end{aligned} \tag{1}$$

As in the main text, when no condition is specified in the average $\langle \cdot \rangle$, we refer to the mean conditioned on the orientation $\Theta = \theta$ prior to a tumble event. Since the mean of the increments dB and dN vanishes, we have:

$$m^1_{\Delta t}(\theta)[\Theta] = \langle 2\max(dB, -dN) \rangle/\Delta t. \tag{2}$$

Next, we investigate the maximum function in cases when a tumble occurs or does not occur and when there is rightward ($dN < 0$) or leftward ($dN > 0$) tumbling. We obtain

$$\langle 2\max(dB, -dN) \rangle/\Delta t \approx 2(1-\lambda\Delta t)0.5\frac{\langle |dB| \rangle}{\Delta t} + 2\lambda 0.5 \langle |\beta| \rangle + 2\lambda 0.5 \langle dB \rangle. \tag{3}$$

Here, the first term on the right-hand side represents the case when no tumble occurs and when $dB > 0$. This happens with probability $0.5(1-\lambda\Delta t)$ since the two increments are assumed to be uncorrelated. The second term on the right-hand side represents a rightward tumble ($dN < 0$), where we assumed $|dB| < |dN|$ because typically the tumble angles are much larger than the thermally induced angular displacements. The third term appears for $dN > 0$. However, as before, the mean of the Gaussian increment vanishes. Hence, we finally obtain:

$$m^1_{\Delta t}(\theta)[\Theta] = (1-\lambda\Delta t)\langle |dB| \rangle/\Delta t + \lambda \langle |\beta| \rangle. \tag{4}$$

Similarly, we determine the third moment:

$$m^3_{\Delta t}(x)[\Theta] = \langle 2\max(dN^3 + dB^3, -3dN^2 dB - 3dB^2 dN) \rangle/\Delta t \tag{5}$$

$$\approx (1-\lambda\Delta t)\langle |dB|^3 \rangle/\Delta t + \lambda \langle |\beta|^3 \rangle + 6\lambda\Delta t D_{\text{rot}} \langle |\beta| \rangle. \tag{6}$$

The first term in the second line refers to a situation without tumbling such that the second argument of the maximum function in the first line vanishes. The second term occurs for leftward tumbling ($dN > 0$), again assuming $|dB| < |dN|$. Finally, for rightward tumbling ($dN < 0$), the second argument of the maximum function is larger than the first and only the last term $3dB^2 dN$ has a non-zero average.
