# Supplementary material for: Inferring the Chemotactic Strategy of P. putida and E. coli Using Modified Kramers-Moyal Coefficients
Source: PLoS Comput Biol. 2017 Jan 23;13(1):e1005329. doi: 10.1371/journal.pcbi.1005329 (PMC5293273; doi:10.1371/journal.pcbi.1005329)
Supplement: S2 Text — (PDF) [file pcbi.1005329.s002.pdf]

S2. Moments of the tumble angle distribution of P.*putida*

In the main text we introduced the tumble angle distribution of P.*putida* as $P_{\text{put}}(|\beta|) = \frac{1}{\mathcal{N}}\big(e^{-(\pi-|\beta|)/\Delta\beta} + C\big)$. The normalization constant is calculated to $\mathcal{N} = \Delta\beta[1 - \exp(-\pi/\Delta\beta)] + C\pi$. For the n-th moment one obtains:

$$\langle|\beta|^n\rangle = \frac{1}{\mathcal{N}}\left[\frac{1-\exp(-\pi/\Delta\beta)}{\Delta\beta^{n+1}}(-1)^n n! + \Delta\beta^{n+1}\sum_{i=1}^{n}(-1)^{n-1}\frac{n!}{i!}(\pi/\Delta\beta)^i + \frac{C}{n+1}\pi^{n+1}\right] \quad (1)$$
