# Supplementary material for: Inferring the Chemotactic Strategy of P. putida and E. coli Using Modified Kramers-Moyal Coefficients
Source: PLoS Comput Biol. 2017 Jan 23;13(1):e1005329. doi: 10.1371/journal.pcbi.1005329 (PMC5293273; doi:10.1371/journal.pcbi.1005329)
Supplement: S3 Text — In this appendix, two sample trajectories are presented and used to discuss the difference between heuristic and systematic tumble recognizer. (PDF) [file pcbi.1005329.s003.pdf]

S3. Trajectory analysis

In the main text we explained that heuristic tumble recognizers require threshold parameters to identify tumble events. Depending on their values, tumble events are recognized or not. Here we illustrate this fact by inspecting two particular trajectories.

Fig. 1(a) shows a trajectory of E.*coli* differently processed. The gray triangles refer to the original recorded data points. Every 10th data point is marked brown, which corresponds to a time step of $\Delta t = 0.5\,\mathrm{s}$ used in our inference method. The colored points are determined by a running average over five data points and used in the heuristic tumble recognizer. The respective speed values are indicated with the same color on the right. By visual inspection of the trajectory, one detects a single tumble event. From the sharp directional change of the brown curve, our systematic tumble recognizer would come to the same result. However, from the the speed track of the smoothed trajectory [see Fig. 1(a), right], a heuristic tumble recognizer might detect one, two, or even three tumbles depending on the threshold depth of a speed minimum (see Appendix [S5. Heuristic tumble recognizer]).

In Fig. 1(b) we show another example. While visual inspection and the brown curve would identify two tumble events, the heuristic tumble recognizer applied to the smoothed data detects between one and four tumbles depending on the speed threshold parameters. Recognizing all tumbles correctly is important since their number directly affects the overall tumble rate.

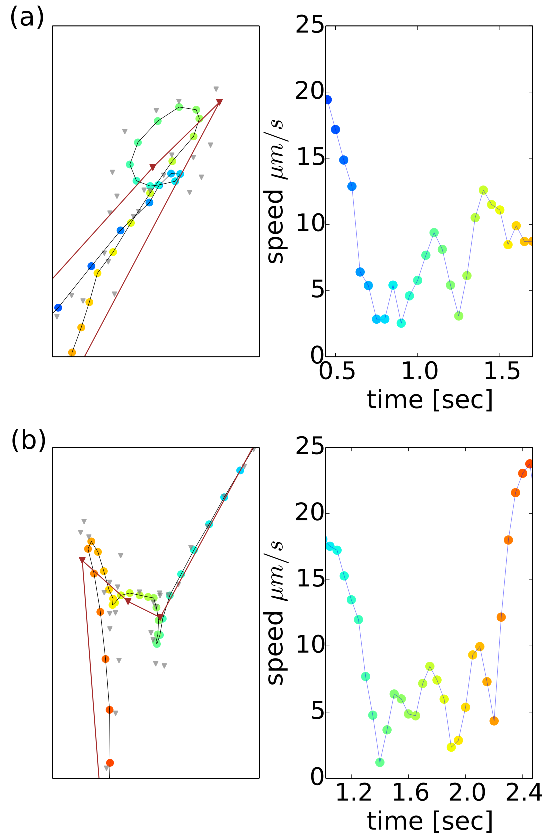

Figure 1: **Two example trajectories of E.*coli* and their assigned speed tracks.** Left: Original data points in gray, every 10th point in brown. Colored points are obtained by a running average over five data points of the original trajectory. Colors indicate time. Right: Speed tracks. Visual inspection of the trajectories and the brown curve suggest one (a) and two (b) tumble events. The speed tracks give up to three (a) or four (b) tumbles depending on the threshold value.
