# Supplementary material for: Inferring the Chemotactic Strategy of P. putida and E. coli Using Modified Kramers-Moyal Coefficients
Source: PLoS Comput Biol. 2017 Jan 23;13(1):e1005329. doi: 10.1371/journal.pcbi.1005329 (PMC5293273; doi:10.1371/journal.pcbi.1005329)
Supplement: S4 Text — The bootstrap method is presented, which allows to determine the variances of the inferred parameters from one set of trajectories. (PDF) [file pcbi.1005329.s004.pdf]

S4. Bootstrap method

In order to obtain reliable standard deviations of the parameters inferred with our CM method, one could perform more experiments. Then, one would repeat the inference for each new set of trajectories and calculate the standard deviation of the inferred set of parameters. An alternative to this time consuming approach is offered by the so-called *bootstrap method* [1]. Its main idea is to create synthetic sets of trajectories from the original one instead of recording new trajectories experimentally.

To this end, we call the set of N experimental trajectories $T_0 := \{t_1, \ldots, t_N\}$. Now, we sample K new sets of trajectories T_i with $i = 1 \ldots K$, each with N elements, and define $T_i := \{\tilde{t}_1, \ldots, \tilde{t}_N\}$, where each $\tilde{t}_j$ is sampled with the same probability out of the experimental trajectories of T_0. By doing so, some of the trajectories of T_0 may appear multiple times in the sets T_i, $i = 1 \ldots K$, others will not appear at all. Then, we perform the parameter inference for each new set T_i and obtain K sets of parameters, which we use to calculate the mean and the variance of the parameters. The standard deviation, is plotted as error bars in the graphs for λ and $\langle|\beta|\rangle$ in the main text. Typically we used $K = 10$ to determine the error bars.

References

[1] B. Efron and R.J. Tibshirani, *An introduction to the bootstrap*, 1994, CRC press
