# Supplementary material for: Inferring the Chemotactic Strategy of P. putida and E. coli Using Modified Kramers-Moyal Coefficients
Source: PLoS Comput Biol. 2017 Jan 23;13(1):e1005329. doi: 10.1371/journal.pcbi.1005329 (PMC5293273; doi:10.1371/journal.pcbi.1005329)
Supplement: S5 Text — Details on the heuristic tumble recognizer are provided. In particular, we explain the threshold parameters, needed to determine tumble events. (PDF) [file pcbi.1005329.s005.pdf]

S5. Heuristic tumble recognizer

The tumble recognition algorithm characterizes tumbles based on variations in speed and turn rate as described in [1] and [2]. For the speed it first identifies local minima in the speed v at time points $t_{\min}$. The two adjacent maxima $t_{1,2}$ are then used to compute the depth of the minimum $\Delta v = \max\left[v(t_1) - v(t_{\min}), v(t_2) - v(t_{\min})\right]$. If the relative depth satisfies $\frac{\Delta v}{v(t_{\min})} > \alpha$ with $\alpha = 1$, the cell is considered to be tumbling for the time, where $v(t) \leq v(t_{\min}) + 0.45\,\Delta v$. For the turn rate we only consider the absolute value of the time series $\omega = |\omega|$. Local maxima are identified at times $t_{\max}$ with adjacent minima at times $t_{1,2}$. If the total angular deviation between t_1 and t_2 satisfies $\sum_t \Delta\theta > \beta\sqrt{D_r(t_2 - t_1)}$, with $\beta = 3.5$ and $D_r = 0.1\,\mathrm{rad}^2\,\mathrm{s}^{-1}$, the cell is considered to be tumbling for the times where $|\omega(t_{\max}) - \omega(t)| \leq 0.85\,\Delta\omega$. The relative change in turn rate is defined in full analogy to the speed case by $\Delta\omega = \max\left[\omega(t_{\max}) - \omega(t_1), \omega(t_{\max}) - \omega(t_2)\right]$. Only one of the criteria – speed or turn rate – is sufficient for a time point to be recognized as a tumble.

References

[1] J.-B. Masson, G. Voisinne, J. Wong-Ng, A. Celani, and M. Vergassola, *Noninvasive inference of the molecular chemotactic response using bacterial trajectories*, PNAS, 109 (2012), pp. 1802–1807

[2] M. Theves, J. Taktikos, V. Zaburdaev, H. Stark, and C. Beta, *A bacterial swimmer with two alternating speeds of propagation*, Biophys J., 105 (2013), pp. 1915–1924
