# Supplementary material for: Inferring the Chemotactic Strategy of P. putida and E. coli Using Modified Kramers-Moyal Coefficients
Source: PLoS Comput Biol. 2017 Jan 23;13(1):e1005329. doi: 10.1371/journal.pcbi.1005329 (PMC5293273; doi:10.1371/journal.pcbi.1005329)
Supplement: S6 Text — We show that our approach is valid for run-time distributions, which have a finite first moment. (PDF) [file pcbi.1005329.s006.pdf]

S6. Moments of shot noise with non-exponential run times

In Eq. (10) we give a formula for the moments of shot-noise. We here extend this formula for shot noise with a non-exponential distribution for the run times T. The number of shots in a given time interval $I_t = [t, t + \Delta t]$ is independent of their amplitude. Hence, we obtain Wald's equation:

$$\langle |dN|^k \rangle = \langle Q_{I_t} \rangle \langle |\beta|^k(\theta) \rangle \ , \tag{1}$$

where Q_{I_t} represents the number of shots within I_t. Now, Blackwell's theorem states that [1]:

$$\lim_{t \to \infty} \langle Q_{I_t} \rangle = \frac{\Delta t}{\langle T \rangle} \ . \tag{2}$$

The limit $t \to \infty$ imposes a stationary state, in which the expectation for the number of shots only depends on Δt. In our experiments, bacteria have swum at least seven minutes before we image them and during which they perform hundreds of tumbling events. Therefore, we can assume Eq. (2) to hold with great precision without taking the limit $t \to \infty$. Since $\langle T \rangle = \lambda^{-1}$, we recover Eq. (10) from the main text.

References

[1] D. Blackwell et al. *A renewal theorem*, Duke math. J., 15 (1948), pp.145–150
