# Supplementary figures and images for: Inferring the Chemotactic Strategy of P. putida and E. coli Using Modified Kramers-Moyal Coefficients
Source: PLoS Comput Biol. 2017 Jan 23;13(1):e1005329. doi: 10.1371/journal.pcbi.1005329 (PMC5293273; doi:10.1371/journal.pcbi.1005329)

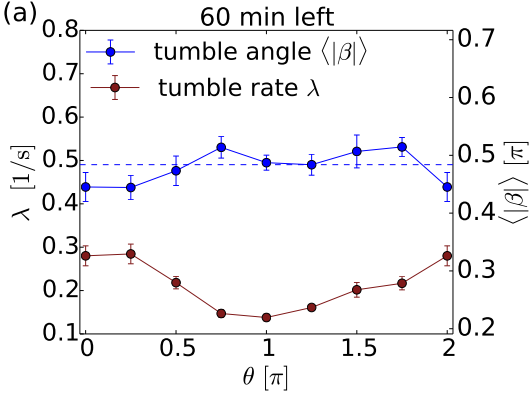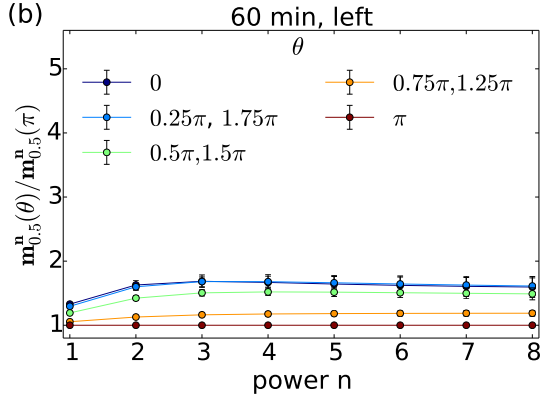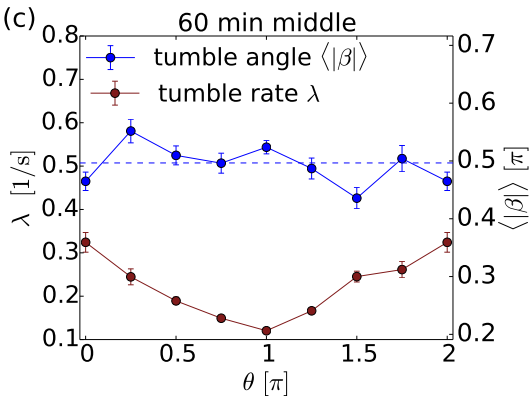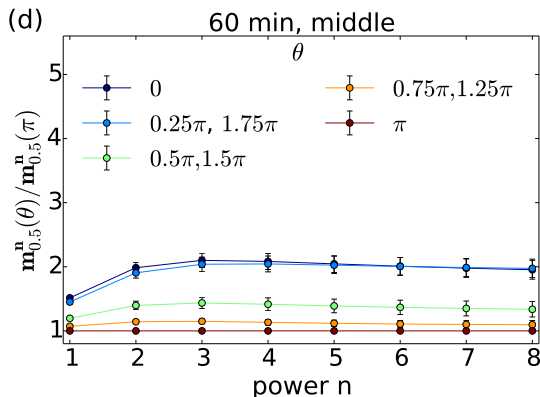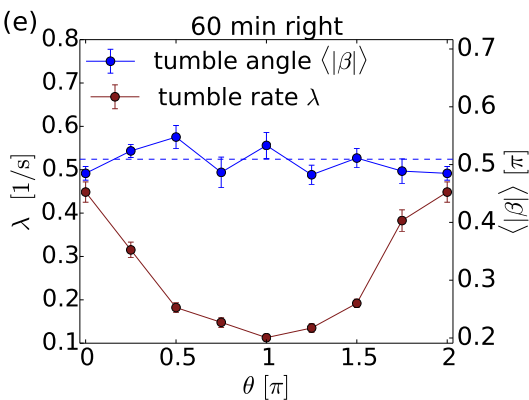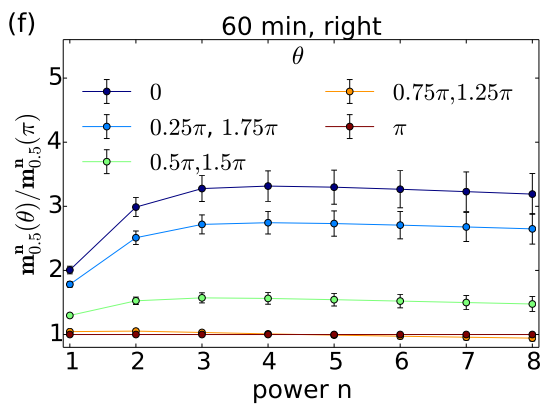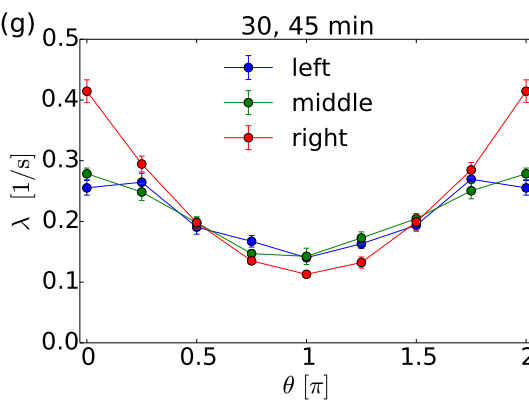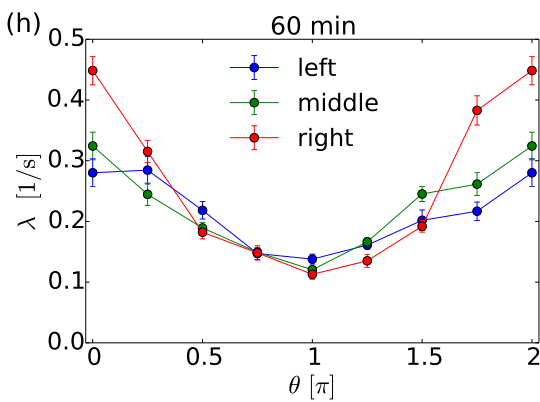

Supplement: S1 Fig — Tumble rate, mean tumble angle, and CM ratios for the late trajectories of E. coli in the left, middle, and right part of the channel are provided. (PDF) [file pcbi.1005329.s007.pdf]

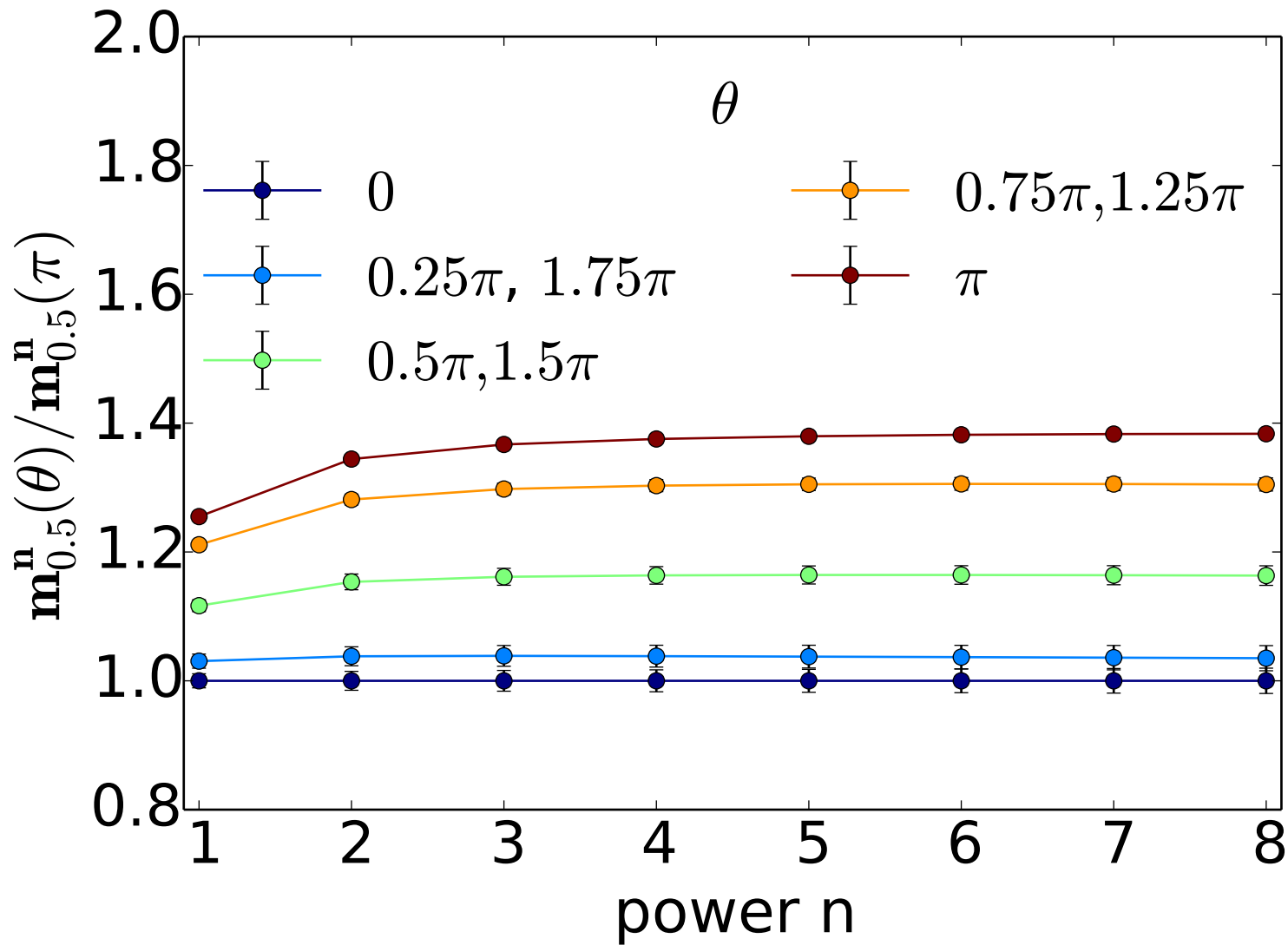

Supplement: S2 Fig — (PDF) [file pcbi.1005329.s008.pdf]

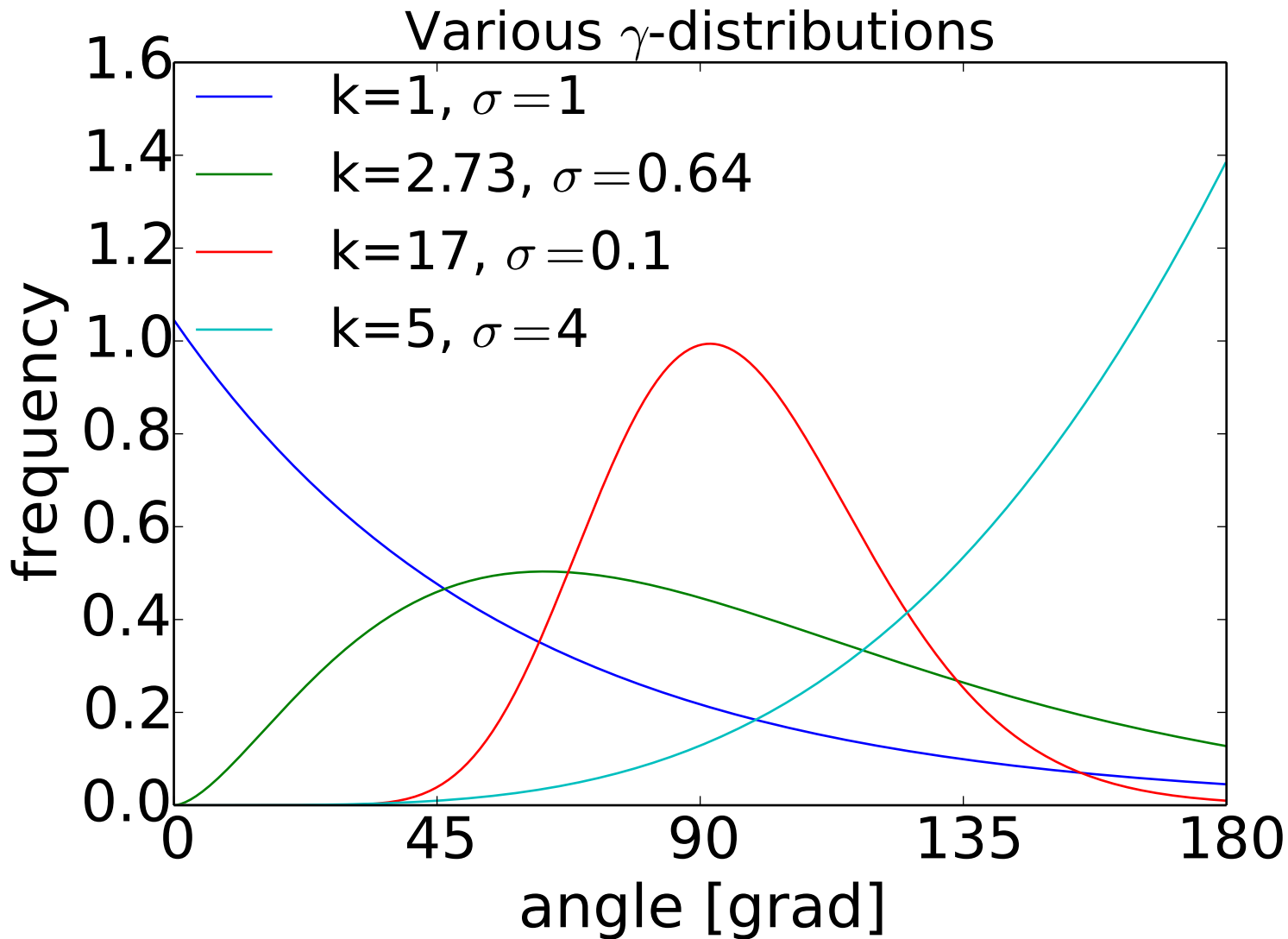

Supplement: S3 Fig — (PDF) [file pcbi.1005329.s009.pdf]

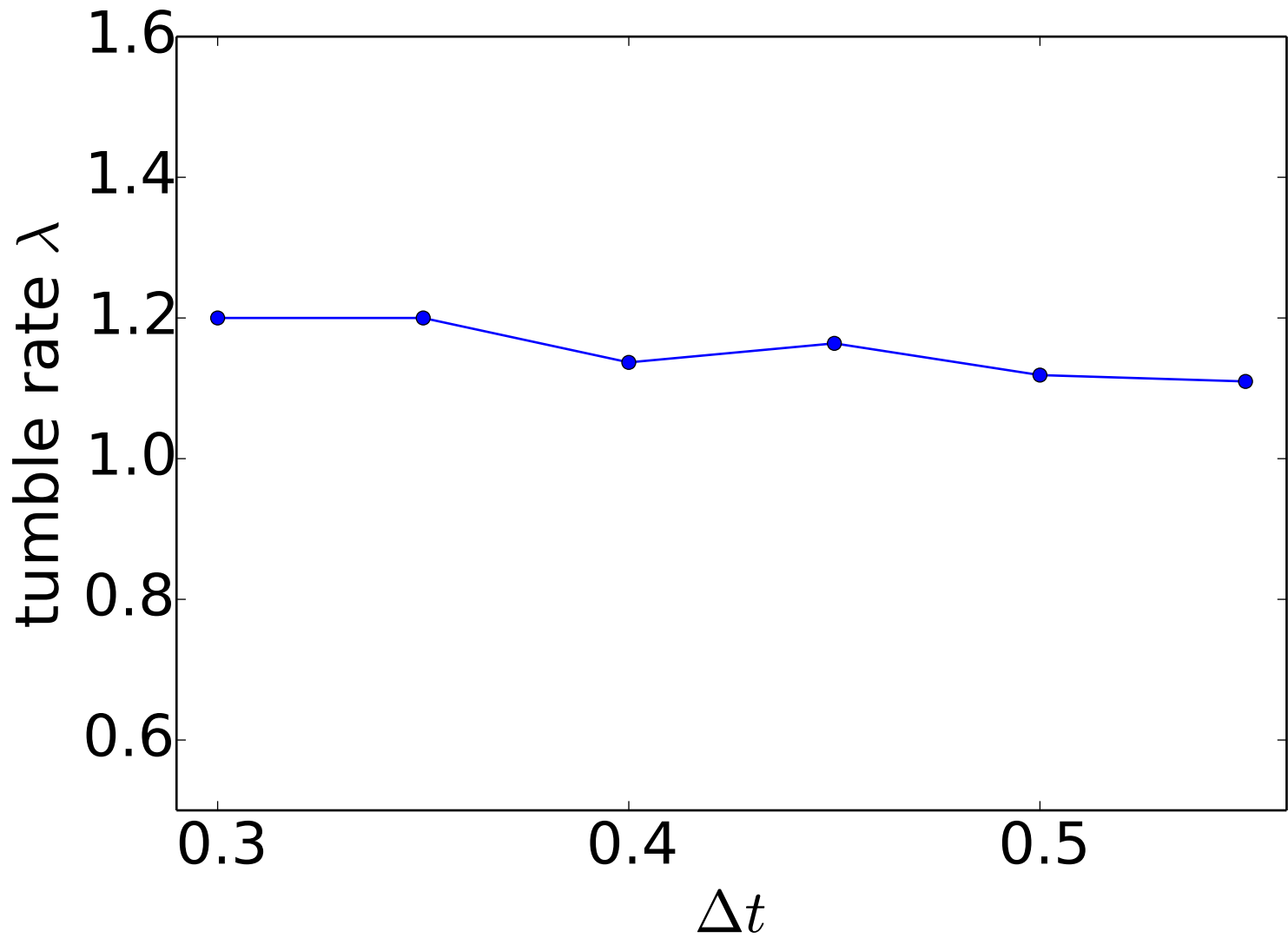

Supplement: S4 Fig — (PDF) [file pcbi.1005329.s010.pdf]
